# TCL1A+ B cells predict prognosis in triple-negative breast cancer through integrative analysis of single-cell and bulk transcriptomic data

**DOI:** 10.1515/biol-2022-0707

**Published:** 2023-09-30

**Authors:** Peifeng Hou, Yang Luo, Ningzi Wu

**Affiliations:** Department of Oncology, Fujian Medical University Union Hospital, Fuzhou, Fujian, 350001, China; Fujian Key Laboratory of Translational Cancer Medicine, Fuzhou, Fujian, 350000, China; Fujian Medical University Stem Cell Research Institute, Fuzhou, Fujian, 350000, China

**Keywords:** triple-negative breast cancer, scRNA-seq, TCL1A+ B, prognosis

## Abstract

Triple-negative breast cancer (TNBC) is an aggressive subtype with limited treatment options and high mortality rates. It remains a prevailing clinical need to distinguish whether the patient can benefit from therapy, such as chemotherapy. By integrating single-cell and global transcriptome data, we have for the first time identified TCL1A+ B cell functions that are prognostically relevant in TNBC. This finding broadens the perspective of traditional tumor-infiltrating lymphocytes in predicting survival, especially the potential value of B cells in TNBC. Single-cell RNA-seq data from five TNBC patients were collected to identify the association between immune cell populations and clinical outcomes. Functional analysis was according to gene set enrichment analysis using pathways from MsigDB. Subsequently, the gene signature of TCL1A+ B cells based on differential expression genes of TCL1A+ B cells versus other immune cells was used to explore the correlation with tumor microenvironment (TME) and construct a prognostic signature using a non-parametric and unsupervised method. We identified TCL1A+ B cells as a cluster of B cells associated with clinical outcomes in TNBC. Functional analysis demonstrated its function in B cell activation and regulation of immune response. The highly enriched TCL1A+ B cell population was found to be associated with a thermal TME with anti-tumor effects. A high abundance of TCL1A+ B cell population is positively correlated with a favorable therapeutic outcome, as indicated by longer overall survival. The present study suggests that TCL1A+ B cells play a key role in the treatment and prognostic prediction of TNBC, although further studies are needed to validate our findings. Moreover, the integration of transcriptome data at various resolutions provides a viable approach for the discovery of novel prognostic markers.

## 1 Introduction

Triple-negative breast cancer (TNBC) is defined as a kind of breast cancer with the absence of estrogen receptor (ER), progesterone receptor, and human epidermal growth factor receptor 2 (HER-2). Because of the lack of molecular targets of therapeutic agents, therapeutic options are limited for TNBC patients [1,2]. Chemotherapy is currently the primary established treatment option for patients with TNBC that significantly improve the prognosis of TNBC patients [3]. Many patients will receive neoadjuvant chemotherapy before surgery so as to reduce the size of the primary lesion and lower the tumor stage to meet the goal of surgical applicability and improve the rate of surgical breast conservation. Some studies have found that 90% of patients undergoing neoadjuvant chemotherapy experience a reduction in tumor size, and 30% of patients even achieve complete remission [4,5]. EGFR overexpression is common in TNBC, which is directly related to the poor prognosis of patients. Treatment with cetuximab can improve the survival rate of patients, which has a significant effect. For women with TNBC who have a BRCA mutation and whose cancer no longer responds to standard breast cancer chemotherapeutic agents, platinum-based chemotherapeutic drug agents such as cisplatin or carboplatin chemotherapy agents or targeted agents known as poly-ADP-ribose polymerase inhibitors such as Lynparza or Talzenna may be considered. In addition, PD-1/PD-L1 inhibitors have also shown sound therapeutic effects in immunotherapy. However, not all patients can obtain clinical benefits from the chemotherapy; approximately 50% of patients had no pathological complete response [4,6–8]. A novel, better treatment option, and personality clinical intervention strategy are still urgently needed.

With the development of single-cell RNA-seq (scRNA-seq) technology, individual transcriptomic gene expression data can be obtained. It provides new chances to explore the tumor microenvironment (TME) heterogeneity of breast cancer and TNBC and perform the association between immune cell populations and clinical outcomes [9–12]. However, the cohort size and the access to clinical outcomes generally become the limitation of related studies.

The prognosis prediction capability of tumor infiltration lymphocytes, particularly T cells, has been described across multiple indications [13–15]. Recent studies have also demonstrated that higher infiltration of B cells might show a favorable prognosis in non-small cell lung cancer, soft tissue sarcoma, colorectal cancer, and renal cell carcinoma [16–19]. As such, it is unclear whether B cells or B cell subpopulations are beneficial in correlation with clinical outcomes or can be used as a prediction factor in TNBC.

This study integrated the scRNA-seq and bulk RNA-seq data with a bioinformatic method to explore the association between immune cell populations and clinical benefits. We identified TCL1A+ B cells, similar to follicular B cells, which positively correlated to favorable clinical outcomes. Furthermore, we constructed a gene signature to demonstrate the correlation between the TME and the B cell subpopulation and uncover its value in predicting prognosis.

## 2 Materials and methods

### 2.1 Public data collection

The scRNA-seq data of five TNBC patients with primary tumors were collected from the Gene Expression Omnibus database. GSE169246 [9] (PD-L1 blockade-induced temporal single cell dynamics in TNBC), which utilized single-cell RNA- and ATAC-sequencing to examine the dynamics of immune cells in metastatic TNBC patients treated with paclitaxel or its combination with atezolizumab. The investigator evaluated the clinical responses based on radiologic assessments of tumor sizes every 8 weeks according to the Response Evaluation Criteria in Solid Tumors (RECIST, version 1.1) [20]. Notably, after the eighth week of treatment initiation, the final response was defined according to the reaction of patients, including PR (responder, complete response/partial response samples) and SD stable disease, progression disease samples. All five patients received chemotherapy with paclitaxel monotherapy.

Two bulk RNA-seq cohorts were utilized in our study. The first one of 116 TNBC patients and their clinical information were downloaded and extracted from the TCGA-BRCA dataset based on ER, progesterone receptor, and HER2 status. Another bulk RNA-seq data of 319 TNBC patients and corresponding clinical information were collected from the Molecular Taxonomy of Breast Cancer International Consortium (METABRIC) project in the cBioportal database [21]. Similar to the TCGA dataset, ER, progesterone receptor, and HER2 gene information was applied to select TNBC patients.

### 2.2 scRNA-seq data processing

The unique molecular identifier (UMI) matrix, barcode list, and features information of five patients were used as input data into Seurat (v4.1) [22]. We utilized the library size correction method to normalize the raw expression matrix with the NormalizeData function. FindVariableFeatures was applied to find out the highly variable genes (HGVs). Normalized expression data were further scaled with the top 2,000 HGVs and regressed with the percentage of mitochondrial gene counts, UMI counts, and patient and cell cycle scores. To reduce noise and remove the batch effect, we performed principal component analysis with 50 principal components (PCs) and selected the top 30 PCs for the RunHarmony function of the harmony package [23]. “Patient” was the variable to remove during the integration. We next constructed a k nearest neighbor graph based on the harmony space with the FindNeighbor function. The Louvain algorithm was applied to cluster the cells using 0.5 as resolution. Uniform manifold approximation and projection (UMAP) was used with the same harmony spaces for visualization, and the expression level of genes was shown by the FeaturePlot function.

We detected differential expression genes (DEGs) using the FindAllMarkers function to annotate the immune cell clusters. Marker genes annotated the significant cell populations. To further identify the specific group, marker genes between cluster 12 and other clusters and cluster 12 versus cluster 9 were detected by FindMakers function with different idents.

### 2.3 Definition of gene signature

From the results of DEGs between cluster 12 and others, genes with log2FC ≥1 and adjusted *p*-value <0.05 were considered as signature genes of TCL1A+ B cells. To further confirm the uniqueness of the gene signature, we utilized the AddModuleScore function to evaluate the abundance of the above gene signature in each cell and displayed it using the RidgePlot function.

### 2.4 Pathway and immune cell populations analysis

To investigate the function of TCL1A+ B cells in scRNA-seq data, DEGs with log2FC ≥1 and adjusted *p*-value <0.05 were considered significantly differential genes to downstream analysis. The gene symbols were converted into Entrez id using the org.Hs.eg.db package. We analyzed biological processes using the enrichGO function from the clusterProfiler package according to the significant DEGs [24]. The top 30 pathways were displayed by dot plot. In addition, we utilized an R package called fast gene set enrichment analysis (GSEA) (fuse) to calculate the empirical enrichment score null distributions simultaneously for gene sets from C2-CP canonical pathways of MsigDB.

To evaluate the association between TCL1A+ B cells and TME in bulk RNA-seq, we performed the abundance score using the given method with gene signatures of TCL1A+ B cells [25]. The patients from TCGA-TNBC were further classified into two groups based on the median value of abundance score. An abundance score of four immune cell populations, including activated CD4+ T cells, activated CD8+ T cells, effector memory CD4+ T cells, and effector memory CD8+ T cells, and four hot-tumor gene sets were conducted by the given method as well.

Raw counts data of TCGA-TNBC were downloaded using the TCGAbiolinks package, and group information was inherited from the above analysis. We analyzed gene rank by a negative binomial generalized linear model to estimate dispersion and logarithmic fold changes incorporating data-driven prior distributions using DESeq2. GSEA was used to evaluate the normalized enrichment scores of pathways from the Reactome pathway database [26–28].

### 2.5 Survival analysis

The TCGA-TNBC and METABRIC-TNBC described above were used to evaluate the prognostic performance of TCL1A+ B cells. We utilized the GSVA method for gene signature to obtain the abundance score of TCL1A+ B cells. The patients from each cohort were classified into high and low groups according to the median value of the abundance score of the given signature, respectively. The Cox proportional hazards regression model was used to evaluate hazard ratios (HRs). The median overall survival (OS) and survival curves were performed using the Kaplan–Meier method.

### 2.6 Statistical analysis

Statistical analysis was performed as described in the figure legends. Correlation coefficients were calculated by Pearson correlation. Survival was measured by the Kaplan–Meier method. Statistical significance was determined by Cox regression, Wilcoxon test, and Benjamini–Hochberg method.

## 3 Results

### 3.1 Association of immune cell populations with clinical outcome

**Figure 1:**
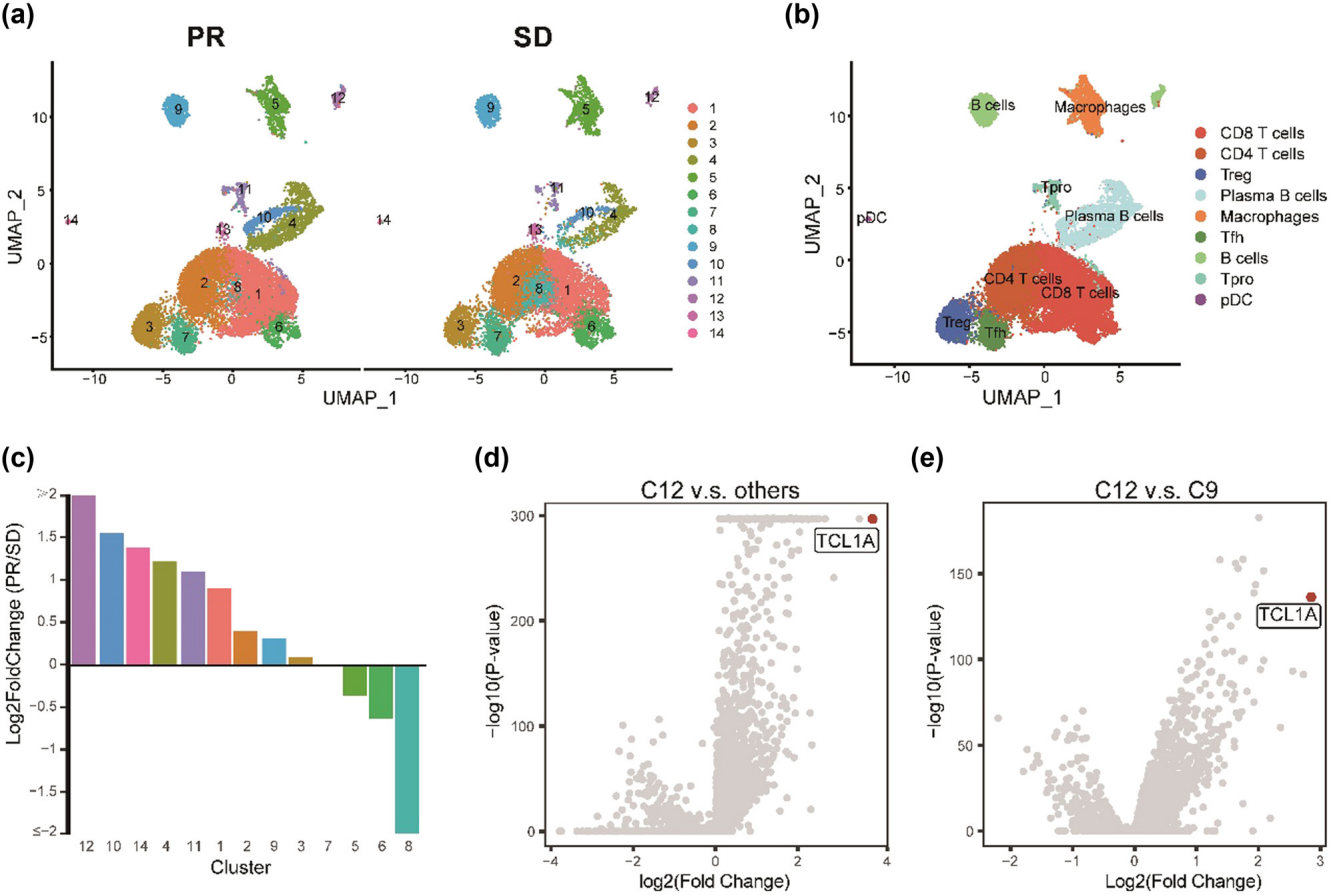
Identification of immune cell populations by scRNA-seq. (a) UMAP plot of immune cell clusters between PR and SD from five TNBC patients. (b) UMAP plot of nine significant immune cell populations. (c) The fold change of the percentages of each of the 14 clusters comparing the PR and SD. (d) Scatter plot of DEGs between cluster 12 and other clusters. (e) Scatter plot of DEGs between cluster 12 and cluster 9. Red point of (d) and (e) represents the top 1 DEG ranked by log2 fold change.

The scRNA-seq data of five TNBC patients were collected from a published study. Two patients were defined as having a partial response, and the others are stable diseases based on RECIST. We utilized the harmony package to integrate all samples and generated 21 cell clusters from 26,384 single cells with Seurat package (Figure 1a). Based on the expression of marker genes, the 14 cell clusters were classified into eight significant immune cell populations (Figure 1b), including CD8+ T cells (CD3D+CD8A+CD4−, clusters 1, 6, 8), CD4+ T cells (CD3D+CD4+CD8A−, cluster 2), regulatory T cells (Tregs) (FOXP3+, cluster 3), follicular helper T cells (Tfh) (CD200+, cluster 7), B cells (CD19+MS4A1+, cluster 9, 12), plasma B cells (MZB1+, cluster 4), macrophage (LYZ+C1QC +, cluster 5), and pDC (LILRA4+, cluster 14).

To investigate the association of immune cells with clinical outcome, we utilized integrative analysis by combining cells from all patients (Figure 1c). Usingtwo-fold differences as a biologically significant threshold, we identified two clusters, cluster 8 and cluster 12, which exceed this criterion. Furthermore, cluster 12 was 2.04-fold higher in the PR group versus the SD group, whereas cluster 8 was 3.74-fold higher in the SD group than in the PR group. We also tested the distribution of cluster 12 at the patient level (Figure S1); the findings provide support for the integrated analysis, indicating that patients in the PR group exhibited a significantly higher proportion of cluster 12, which is associated with a pro-tumoral environment, compared to almost all patients in the SD group. Unfortunately, the statistical analysis cannot be done due to the small size of the samples.

To further annotate cluster 12, we intersected the DEGs between cluster 12 versus other groups and cluster 12 versus another B cell cluster (cluster 9) (Figure 1d and e, Table S1). TCL1A (TCL1 family AKT coactivator A), as the highest expression differential gene in both groups, was used to annotate cluster 12. Cluster 12 is thus named TCL1A+ B cells.

Overall, we identified two clusters; a subset of CD8+ T cells and a subset of B cells were highly related to clinical outcomes in TNBC. In contrast, only the B cell subset, TCL1A+ B cells, positively correlate with PR patients.

### 3.2 Functional exploration of TCL1A+ B cells

**Figure 2:**
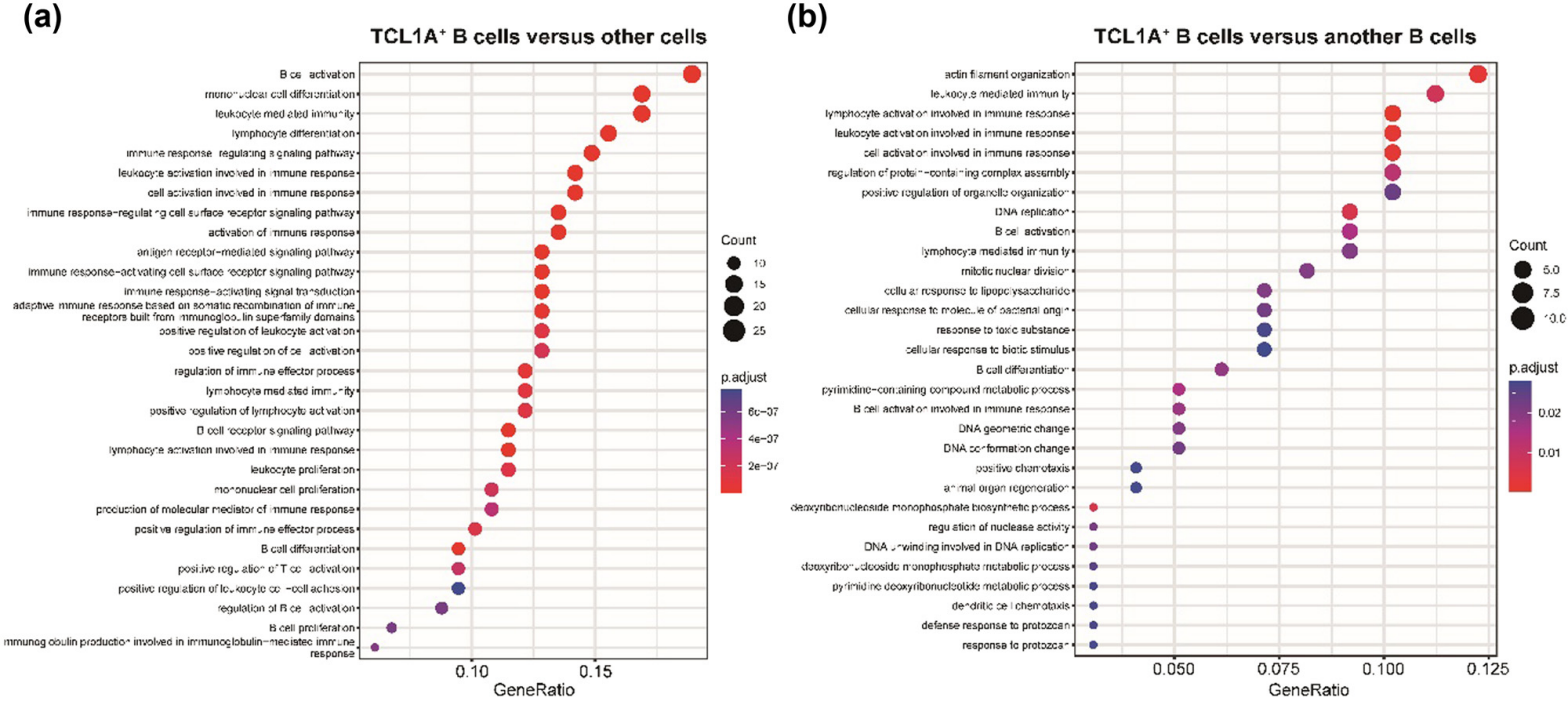
Enrichment biological processes of TCL1A+ B cells. (a) Enrichment biological processes between TCL1A+ B cells and other immune cell populations. (b) Enrichment biological processes between TCL1A+ B cells and another B cell populations.

We investigated the function of TCL1A+ B cells in the above scRNA-seq data. By comparing transcriptomic differences of TCL1A+ B cells versus other immune cell populations, we found that TCL1A+ B cells significantly enriched in B cell-related immune response and regulation of T cell activation biological processes (Figure 2a), such as B cell activation, activation of the immune response, and positive regulation of T cell activation, indicating the association with anti-tumor immune response. Moreover, compared to another B cell population (cluster 9), the proliferation, differentiation, and activation function was more robust in TCL1A+ B cells than in another (Figure 2b).

To explore the potential functional differences of TCL1A+ B cells between the PR and SD groups, we utilized GSEA with pathways from five pathway databases based on gene rank from the two groups. The results showed that TCL1A+ B cells from SD patients are more likely to be enriched in hypoxia, NFAT, and TNF-alpha pathways, correlated with tumor invasion and metastasis, indicating a pro-tumoral environment (Table S3).

### 3.3 Higher TCL1A+ B cells are associated with an anti-TME

**Figure 3:**
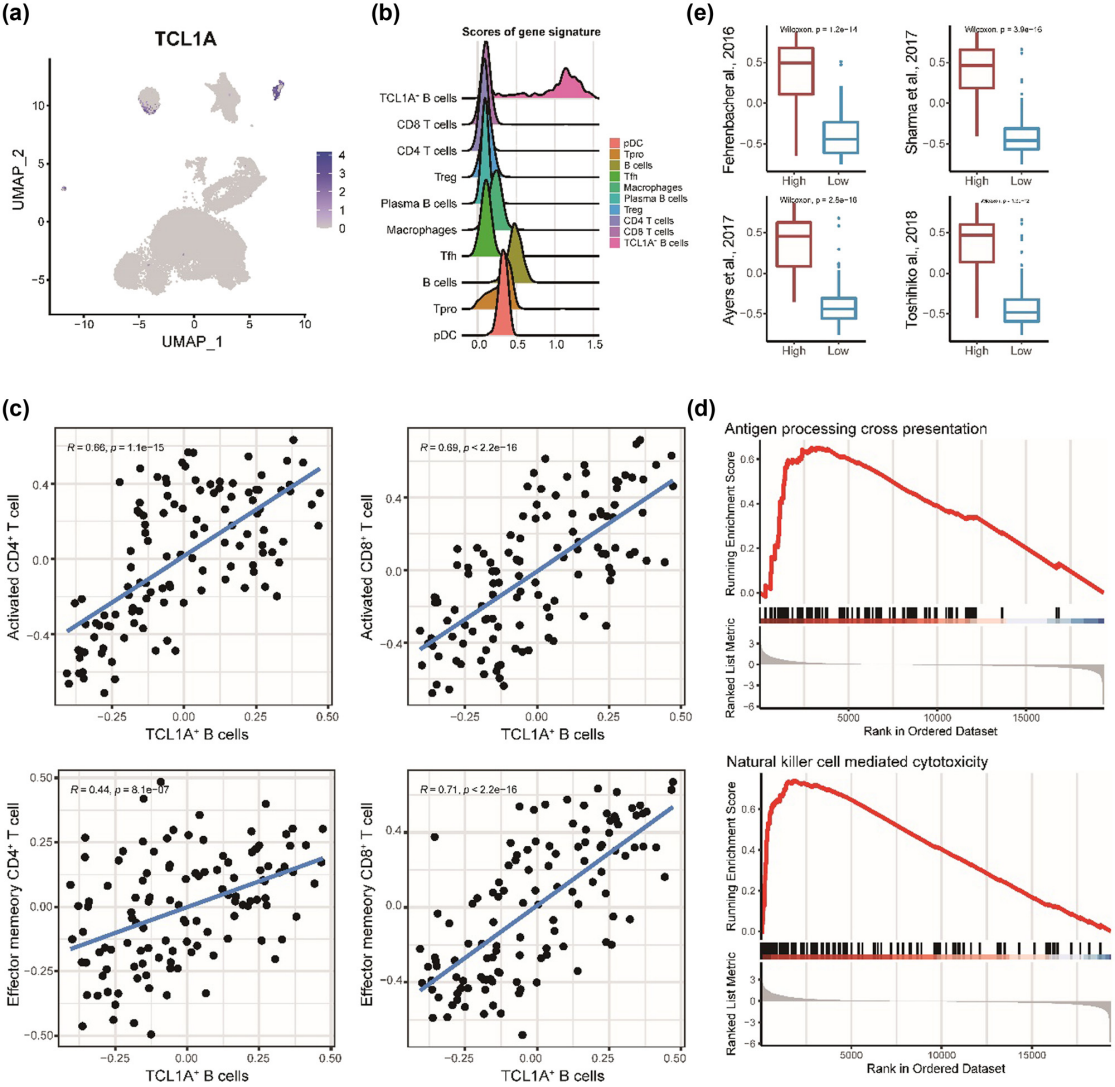
Association between TCL1A+ B cells and TME. (a) Scatter plot of TCL1A expression, color gradation represents the expression level. (b) Ridge plot of the density of the mean expression value of TCL1A+ B cell gene signature in significant cell populations. (c) Scatter plot of correlation between TCL1A+ B cells and activated CD4 + T cells, activated CD8 + T cells, effector memory CD4 + T cells, and effector memory CD8 + T cells. (d) GSEA results of two pathways between two groups. (e) Box plots of four hot-tumor scores in two groups. Two groups represent high abundance of TCL1A+ B cells and low abundance of TCL1A+ B cells with median abundance score as a cut-off.

Despite TCL1A being selected to represent cluster 12, it was expressed by a few cells of another B cell population as well (Figure 3a). Based on previous studies [29,30], the high expression level of BCL6, NEIL1, and MEF2B proposed that our TCL1A+ B cells are more similar to follicular B cells. To perform the abundance of TCL1A+ B cells in bulk transcriptomic data, we first constructed a signature with 165 genes (Table S2). Examining the signature in scRNA-seq data showed that the signature could further distinguish the TCL1A+ B cells from other immune cell populations, especially other B cells (Figure 3b).

To investigate the association between TCL1A+ B cells and tumor environment in the TCGA-TNBC cohort, which is a subset of the TCGA-BRCA cohort, we estimated the abundance of TCL1A+ B cells in each patient through the GSVA score of the gene signature. By correlation analysis, we found that TCL1A+ B cells are strongly positively correlated with activated CD4+ and CD8+ T cells and effector memory CD4+ and CD8+ T cells (Figure 3c), which have played crucial roles in the adaptive immune response. Interestingly, GSEA results showed that antigen processing cross-presentation and natural killer cell-mediated cytotoxicity were enriched in a high group of TCL1A+ B cells (Figure 3d), proposing that innate immune response was promoted, even though antigen processing and presentation was involved in adaptive immune as well. To characterize the TME, we collected four gene sets to represent hot-tumor. The hot-tumor scores were significantly increased in the TCL1A+ B cells high group with all gene sets (Figure 3e). Taken together, our results described that the TME of the high abundance TCL1A+ B cells’ patients is related to hot-tumor with an anti-tumor response.

### 3.4 Higher TCL1A+ B cells are correlated with a favorable prognosis

**Figure 4:**
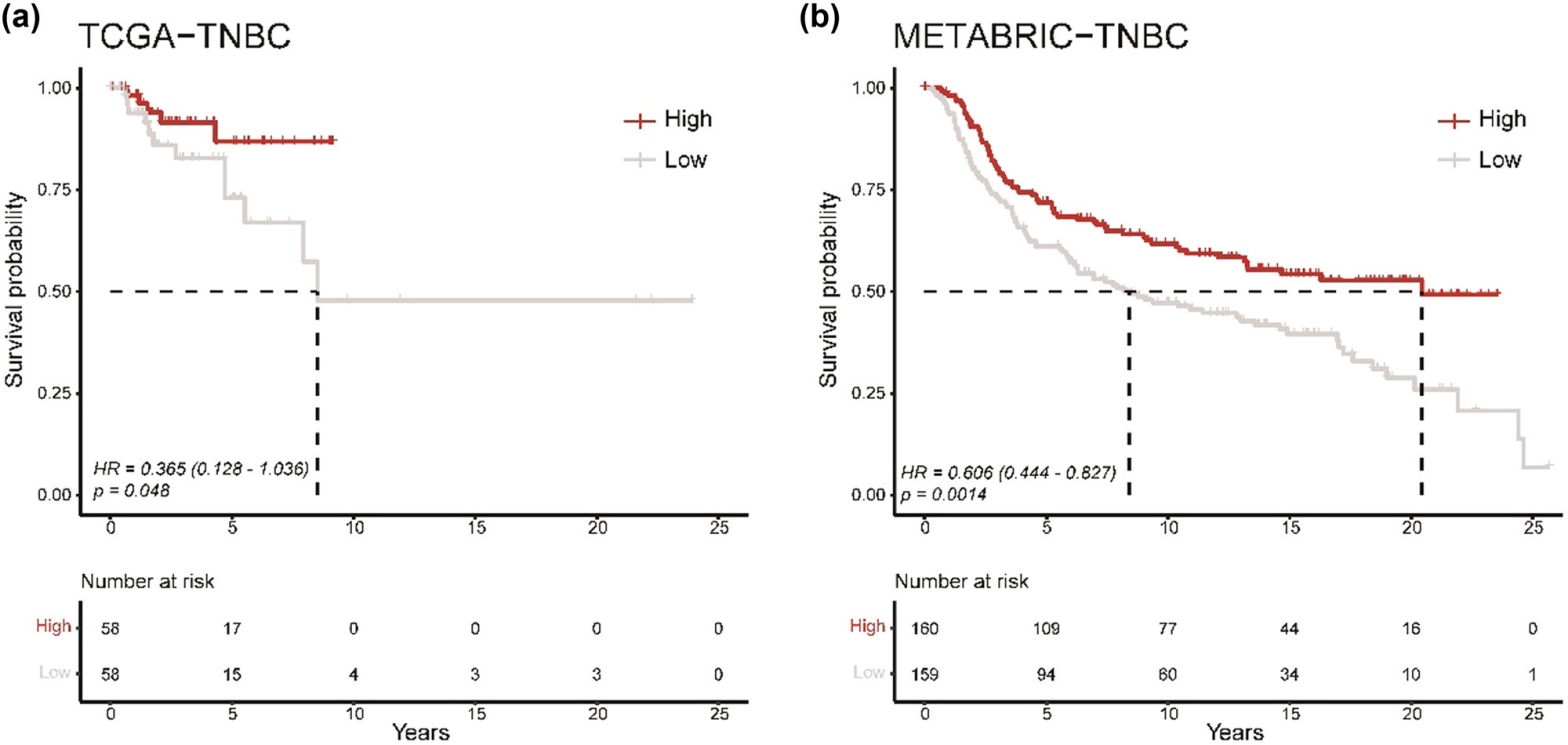
Survival analysis of TCL1A+ B cells: (a) Kaplan–Meier curves of OS to the abundance of TCL1A+ B cells in TCGA-TNBC and (b) METABRIC-TNBC. High represents the high abundance of TCL1A+ B cells, and low means the low abundance of TCL1A+ B cells, with the median abundance score of each cohort as a cut-off.

Next, we utilized the same analysis approach that classified TCGA-TNBC patients into two groups based on the TCL1A+ B cells to evaluate the correlation with OS. The survival analysis showed that OS time in the high abundance group is significantly longer than in the low group (HR: 0.365, 95% CI: 0.128–1.036, *p*-value: 0.048, Figure 4a). However, the number of TNBC patients in TCGA is small (*n* = 116); to further confirm the results, we collected another cohort with 319 TNBC patients. Concordant survival result of METABRIC cohort was observed in the high and low TCL1A+ B cells group (HR: 0.606, 95% CI: 0.444–0.827, *p*-value: 0.0014, Figure 4b). Notably, the median OS time is 2.43-fold longer in the high group (mOS: 20.44 years) than in the low group (mOS: 8.42 years), indicating that the patients who had a higher abundance of TCL1A+ B cells preferred to have a better clinical outcome and longer OS time.

## 4 Discussion

TNBC is an aggressive subtype of breast cancer with limited therapeutic options and high rates of mortality. Identifying patients more likely to obtain clinical benefit from therapy is essential. The improvement of sequencing technology allows for exploring the tumor environment in a single-cell solution. With the clinical information, we can construct an insight between immune cells and outcomes to detect which resistant cell population is related to a favorable clinical outcome. The innovation of this study is to integrate single-cell and global transcriptome data to discover new survival predictive markers, and to reveal the mechanism by which TCL1A+ B cells regulate the TME and affect the survival of TNBC. This provides new ideas for exploring individualized treatment strategies based on TCL1A-B cells in the future.

Our study collected scRNA-seq data from five TNBC patients to construct an immune cell atlas for primary tumors with eight significant resistant cell populations. We further identified a cluster of B cells, annotated based on the common DEG, TCL1A, between the group and other immune cells and the group and another B cell cluster. TCL1A, as a protein-coding gene, is also known as T cell leukemia/lymphoma protein 1A, which has been reported to enhance the phosphorylation and activation of AKT1, AKT2, and AKT3, improve cell proliferation, stabilize mitochondrial membrane potential, and promote cell survival [31–34]. Recently, integrated transcriptomic and epigenetic analysis suggests it also acts as a transcription factor and coregulator of NF-κB p65 in lymphoblastoid cell lines [35]. In Raji Burkitt lymphoma, TCL1A has been connected with TP63 [36]. These findings indicate that TCL1A is essential in multiple biological processes, even in the tumor. Moreover, TCL1A has been proposed as a potential therapeutic target in B cell lymphoma necessary for malignant B cell survival, indicating its association with B cells [37].

Studies have shown the association between B cells and clinical outcomes in solid tumors, such as head and neck squamous cell carcinoma and lung cancer [38,39]. A previous study of the total RNA-seq of flow-sorted samples reported that B cells with TCL1A expression correlated with improved survival time in cervical tumors, suggesting its prediction potential [40]. However, the study of B cells, particularly TCL1A+ B cells, and clinical benefit in TNBC is limited. Based on fold change analysis, we found that TCL1A+ B cells are more than two-fold in PR patients than in SD patients in TNBC. The sample-based analysis revealed that one PR patient had a higher fraction of TCL1A+ B cells than all other SD patients (Figure S1). The results propose that TCL1A+ B cells are associated with favorable clinical outcomes.

A recent review, which discusses various B cell signatures in normal and diseased tissues, has shown that TCL1A can be used as a marker for both naïve B cells and follicular B cells in different tissues [41]. However, our analysis proved that it is more similar to follicular B cells, which generally existed in the germinal center, rather than naïve B cells based on the gene marker of BCL6, NEIL1, and MEF2B [29,30]. The functional analysis of the TCL1A+ B cells showed that it was mainly involved in B cell activation, immune response, and regulation of T cell activation, partially differing from the function of naïve B cells. One major limitation of this study is that the cohort size of scRNA-seq data is limited due to our strict restriction of the sample site, so sample-based analysis does not have significant results. We are also collecting data and planning to reappear the findings with a large cohort in the future.

To further investigate TCL1A+ B cells in bulk RNA-seq, we constructed a gene signature to evaluate the abundance of such cells. With multiple analysis strategies, we found that TCL1A+ B cells are positively correlated with several T cells, for example, activated CD8 T cells, which have been proven to support anti-tumor immune response. The results proposed that innate immune response has been enhanced as well. Interestingly, examination by four hot-tumor gene signatures showed that tumors with a high abundance of TCL1A+ B cells showed hot-tumor characterization, indicating that an immune checkpoint inhibitors-based therapy strategy might be helpful in such patients [42]. Finally, survival analysis in two cohorts demonstrated longer survival time in higher TCL1A+ B cells patients, suggesting its potential to predict prognosis in TNBC.

## 5 Conclusion

According to our study, TCL1A+ B cells as a B cell subset are associated with better clinical outcomes in TNBC patients treated with chemotherapy. By integrating single-cell and global transcriptome data, we found that higher abundance of TCL1A+ B cells was associated with a stronger anti-midstream immune microenvironment and longer OS. These results suggest that TCLIA + B cells play an important role in the prediction of TNBC treatment and prognosis, but further studies are needed to verify our findings. By combining transcriptome data with different segmentation rates, our study reveals a feasible pathway to find novel biomarkers for predicting prognosis.

In our study, we identified an association between TCL1A+ B cells and the clinical outcome of TNEC. Functional analysis showed that TCL1A+ B cells were related to the activation of B cells and immune regulation [2]. In addition, we constructed a gene signature based on differentially expressed genes between TCL1A+ B cells and other immune cells to demonstrate the relationship with the TME, and constructed a prognostic signature using non-parametric and unsupervised methods.

We also found that the OS time of the TCL1A+ B cell high abundance group was significantly longer than that of the low abundance group in TCGA-TNBC and METABRIC-TNBC patients. These data suggest that patients with higher abundance of TCL1A+ B cells tend to achieve better clinical treatment outcomes and longer survival [4]. When studies considered drug efficacy, TCL1A+ B cells were found to also predict clinical response, but this needs further confirmation.

In conclusion, our study fully confirmed the potential therapeutic predictive and prognostic relevance of TCL1A+ B cells in TNBC patients. If validated, this would provide new targets for fine-tuning treatment strategies to improve treatment outcomes.

## Supplementary Material

Supplementary Figure

Supplementary Table 1

Supplementary Table 2

Supplementary Table 3
