# Supplementary material for: TCL1A+ B cells predict prognosis in triple-negative breast cancer through integrative analysis of single-cell and bulk transcriptomic data
Source: Open Life Sci. 2023 Sep 30;18(1):20220707. doi: 10.1515/biol-2022-0707 (PMC10543705; doi:10.1515/biol-2022-0707)
Supplement: Supplementary Figure [file biol-2022-0707-sf.pdf]

# Supplementary material

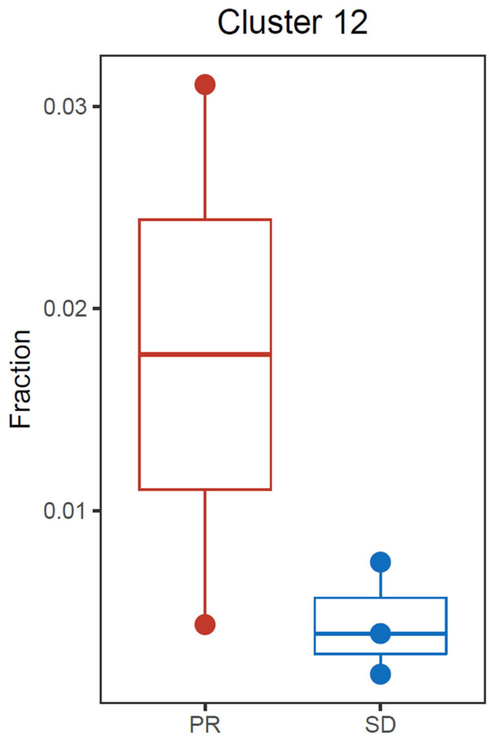


**Figure S1:** Box plot of the fraction of cluster 12 in each sample; Color represents groups (PR or SD).
